# Higher Screening Aldosterone to Renin Ratio in Primary Aldosteronism Patients with Diabetes Mellitus

**DOI:** 10.3390/jcm7100360

**Published:** 2018-10-16

**Authors:** Chia-Hui Chang, Ya-Hui Hu, Kuo-How Huang, Yen-Hung Lin, Yao-Chou Tsai, Che-Hsiung Wu, Shao-Yu Yang, Chin-Chen Chang, Ching-Chu Lu, Kwan-Dun Wu, Vin-Cent Wu

**Affiliations:** 1 Division of Endocrinology and Metabolism, Department of Internal Medicine, Taipei Tzu Chi Hospital, The Buddhist Medical Foundation, Taipei 23142, Taiwan; giahwe@gmail.com (C.-H.C.); huyahui@tzuchi.com.tw (Y.-H.H.); 2 Graduate Institute of Clinical Medicine, College of Medicine, National Taiwan University, Taipei 10002, Taiwan; 3 Department of Urology, National Taiwan University Hospital and National Taiwan University College of Medicine, Taipei 10002, Taiwan; kuohowhuang@gmail.com; 4 Division of Cardiology, Department of Internal Medicine, National Taiwan University Hospital, Taipei 10002, Taiwan; austinr34@gmail.com; 5 Division of Urology, Department of Surgery, Taipei Tzu Chi Hospital, The Buddhist Medical Foundation, Taipei 23142, Taiwan; tsai1970523@yahoo.com.tw; 6 Division of Nephrology, Department of Internal Medicine, Taipei Tzu Chi Hospital, The Buddhist Medical Foundation, Taipei 23142, Taiwan; tcubear@gmail.com; 7 Division of Nephrology, Department of Internal Medicine, National Taiwan University Hospital, Taipei 10002, Taiwan; yangsy@ntuh.gov.tw (S.-Y.Y.); kdwu@ntuh.gov.tw (K.-D.W.); 8 Department of Medical Imaging, National Taiwan University Hospital and National Taiwan University College of Medicine, Taipei 10002, Taiwan; macotocc@gmail.com; 9 Department of Nuclear Medicine, National Taiwan University Hospital and National Taiwan University College of Medicine, Taipei 10002, Taiwan; kelvinlu@ntu.edu.tw; 10 TAIPAI, Taiwan Primary Aldosteronism Investigation (TAIPAI) Study Group, Taipei 10002, Taiwan

**Keywords:** primary aldosteronism, aldosterone to renin ratio, diabetes mellitus, TAIPAI

## Abstract

Accumulated evidence has shown that low renin hypertension is common in patients with diabetic nephropathy. However, the performance of aldosterone to renin ratio (ARR) in primary aldosteronism (PA) patients with diabetes has not been well validated. Here, we report the performance of screening ARR in PA patients with diabetes. The study enrolled consecutive patients and they underwent ARR testing at screening. Then the diagnosis of PA was confirmed from the Taiwan Primary Aldosteronism Investigation registration dataset. Generalized additive model smoothing plot was used to validate the performance of screening ARR in PA patients with or without diabetes. During this study period, 844 PA patients were confirmed and 136 (16.0%) among them had diabetes. Other 816 patients were diagnosed with essential hypertension and used as the control group and 89 (10.9%) among them had diabetes. PA patients with diabetes were older and had a longer duration of hypertensive latency, higher systolic blood pressure and lower glomerular filtration rate than those PA patients without diabetes. The cut-off value of ARR in the generalized additive model predicting PA was 65 ng/dL per ng/mL/h in diabetic patients, while 45 ng/dL per ng/mL/h in non-diabetic patients. There was a considerable prevalence of diabetes among PA patients, which might be capable of interfering with the conventional screening test. The best cut-off value of ARR, more than 65 ng/dL per ng/mL/h in PA patients with diabetes, was higher than those without diabetes.

## 1. Introduction

Primary aldosteronism (PA), one of the most frequent endocrine causes of secondary hypertension, accounts for 3.9% of patients with stage 1 hypertension and 11.8% of patients with stage 3 hypertension [1]. Surgery is indicated for unilateral adrenal diseases like aldosterone-producing adenoma (APA) or unilateral adrenal hyperplasia (UAH). The hypertension cure rate after adrenalectomy is 30–60% [2,3]. Therefore, it raises the crucial importance of early diagnosis of aldosteronism.

Early detection and management of PA not only decreases cardiovascular (CV) morbidity and mortality but also dramatically improves hypertensive remote organ injury via target treatments [4,5,6]. Recent clinical practice guidelines recommend screening for PA with aldosterone to renin ratio (ARR) in hypertensive patients [2,7]. However, many physiological conditions can interfere with the accuracy of ARR. Understanding how certain factors influence ARR (e.g., ARR increases with age and severity of renal impairment) is important so we can increase the diagnostic accuracy of ARR for PA, and avoid unnecessary further tests [2].

Diabetes mellitus (DM), one of the most common diseases (with an estimated total of 366 million people diagnosed worldwide in 2011 and predictions to reach 552 million by 2030), is a leading cause of death in many countries [8]. Previous studies reported that DM is more prevalent in patients with PA compared to patients with essential hypertension (EH) [9]. In addition, many studies also indicated that there is a high prevalence of PA among DM patients with resistant hypertension [10,11].

A previous study observed that patients with DM are more likely to have low plasma renin activity (PRA) [12]. It is probably the renin-angiotensin-aldosterone system (RAAS) that is affected by DM status [13,14]. The best cut-off value of ARR in PA patients with DM may be different from those without DM while ARR is used as a screening test. To our knowledge, there has been no study evaluating the effects of concomitant DM on ARR cut-off level. Therefore, we conducted this observational study and determined the best cut-off value of screening ARR for the diagnosis of PA in patients with DM.

## 2. Materials and Methods

### 2.1. Ethics Statement

The study was approved by the institutional review board of National Taiwan University Hospital (Taipei, Taiwan) (No. 200611031R). All protocol and procedures complied with the standards of the Declaration of Helsinki. Consent has been obtained from each patient or subject after full explanation of the purpose and nature of all procedures used.

### 2.2. Subjects

All patients were registered in the Taiwan Primary Aldosteronism Investigation (TAIPAI) between June 2008 and January 2015 [15,16]. This retrospective study enrolled patients who were referred to the TAIPAI study group that performed ARR test for screening case detection of possible PA during the study period, including two medical centres, three affiliated hospitals and two regional hospitals in several cities in Taiwan [17,18]. Patients with other causes of secondary hypertension, including renovascular hypertension, Cushing’s syndrome, hyperthyroidism and pheochromocytoma, were excluded from this study [19]. Patients were instructed to maintain their usual sodium intake during the study, and adherence was assessed by measuring urinary sodium excretion at each visit. All anti-hypertensive medications were discontinued for at least 2 weeks except diuretics which was/were discontinued for at least 4 weeks before the screening test. Doxazosin and/or diltiazem were administered to control markedly high blood pressure (BP) when required [20].

### 2.3. Diagnosis and Further Lateralisation of PA

The diagnosis of PA was established in hypertensive patients on the basis of the following criteria [21,22].

#### 2.3.1. Confirmation

Fulfilment of the following three conditions confirmed a diagnosis of PA:

1. autonomous excess aldosterone production evidenced with a 24-h urinary aldosterone level (Uald-24 h) more than 20.3 µg [14];
2. TAIPAI score greater than 60% [16];
3. post-saline loading plasma aldosterone concentration (PAC) > 10 ng/dL or PAC/PRA > 35 ng/dL per ng/mL/h shown in a post-captopril/losartan test or PAC > 6 ng/dL indicated by a fludrocortisone suppression test [7].

#### 2.3.2. Subtype Identification

APA was identified on the basis of the following:

1. adrenal adenoma evidenced with a CT scan for pre-operative evaluation; and
2. lateralisation of aldosterone secretion at adrenal venous sampling (AVS) or during dexamethasone suppression NP-59 SPECT/CT [23]; and
3. pathologically proven adenoma after an adrenalectomy for those who undergo surgery; and
4. subsequent evidence of either a complete or partial cure of hypertension [24].

Idiopathic hyperaldosteronism (IHA) was distinguished on the basis of the following:

1. evidence of bilateral diffuse adrenal enlargement indicated on CT scan; or
2. non-lateralisation of aldosterone secretion at AVS or during dexamethasone suppression NP-59 SPECT/CT [23]; or
3. evidence of diffuse adrenal cell hyperplasia reported in pathology studies for those undergoing surgery.

### 2.4. Definitions of General Parameters and Laboratory Data

We collected information on the following clinical parameters and laboratory data from medical records: age, gender, body mass index (BMI), duration of hypertension, systolic blood pressure (SBP), diastolic blood pressure (DBP), heart rate (HR), number of anti-hypertensive medications, comorbidities, adrenal function tests, glomerular filtration rate (GFR), serum potassium levels and pathological findings. BP was measured using a sphygmomanometer during the initial evaluation of PA in the outpatient department. Using office BP measurement, hypertension was defined as SBP ≥ 140 mmHg and/or DBP ≥ 90 mmHg and/or use of anti-hypertensive medication(s) [25]. Blood samples measuring PAC and PRA were obtained in the sitting position with anti-hypertensive medication discontinuation or modification in the first drawn blood. At the same time, we also collected the preoperative serum potassium level and estimated glomerular filtration rate (eGFR), presented as the chronic kidney disease epidemiology collaboration equation (CKD EPI). Diabetic mellitus (DM) was defined as HbA1C ≥ 6.5%, fasting plasma glucose ≥ 126 mg/dL, 2-h plasma glucose ≥ 200 mg/dL, random plasma glucose ≥ 200 mg/dL with classic symptoms of hyperglycaemia or the use of anti-diabetic medications [26].

### 2.5. Functional Survey

PAC was measured by radioimmunoassay using a commercial kit (Aldosterone Maia Kit, Adaltis Italia S.P.A., Bologna, Italy). PRA was measured by the generation of angiotensin I in vitro using a commercially available radioimmunoassay kit (DiaSorin, Stillwater, MN, USA) [14,21].

### 2.6. Statistical Analyses

Results were expressed as the mean and the standard deviation (SD). Log transformation was applied for skewed distributions, such as PAC, PRA and ARR. Univariate analyses were performed using independent *t*-tests. Chi-square tests was used for the comparison of two proportions. Receiver operating characteristic (ROC) curve was used to evaluate the cut-off value of screening ARR in PA patients with or without DM. In order to display the implications of ARR for individual patients, a generalized additive model (GAM) (with spline) incorporating the subject-specific (longitudinal) random effects were plotted and adjusted for other clinical parameters to predict the possibility of PA [27,28]. Simple and multiple generalised additive models (GAMs) were fitted to detect nonlinear effects of continuous covariates and identify the appropriate cut-off point(s) for discretising continuous covariates, if necessary, during the stepwise variable selection procedure. We defined the optimal cut-off value as log odd equals to zero. The vgam function (with the default values of smoothing parameters) of the VGAM package [21,29] was used to fit GAMs for the binary responses in R software, version 2.8.1 (Free Software Foundation, Inc., Boston, MA, USA).

Statistical significance was defined as *p* < 0.05. Statistical analyses were performed with MedCalc Statistical Software version 16.4.3 (MedCalc Software bvba, Ostend, Belgium; https://www.medcalc.org; 2016) and R software, version 2.8.1 (Free Software Foundation, Inc., Boston, MA, USA).

## 3. Results

### 3.1. Clinical Characteristics of PA and EH Patients with DM or without DM

During this period, a total of 1660 hypertensive patients (mean age 51.2 ± 13.8, male 50%) were enrolled in this study, and 844 patients (mean age 52.5 ± 12.4 years, male 46%) among them were confirmed to have PA; the other patients were diagnosed as ‘essential hypertension’ (EH), and used as the control group in some comparisons. The comparison between PA patients and EH patients was shown in Table S1. Among PA patients, 136 (16%) had DM and 625 (74%) patients had APA. Table 1 summarises the demographic and clinical data of the 844 PA patients at enrolment. PA patients with DM were older; had a higher percentage of men, higher BMI, longer duration of hypertensive history, higher SBP, higher prevalence of coronary artery disease (CAD), lower eGFR and used more anti-hypertensive medications than those PA patients without DM.

The clinical characteristics of 225 DM patients with and without PA were shown in Table 2. In our diabetic cohort, there was no type 1 DM. There were 816 EH patients and 89 (10.9%) among them had DM. The clinical characteristics of controlled EH patients with DM or without DM were shown in Table S2.

### 3.2. Effects of DM Status on ARR

Dot plots for log-transformed PAC, PRA and ARR in PA patients with DM were compared to those without DM (Figure S1). Log-transformed PAC, PRA and ARR were not statistically different between the two groups. The result of ROC curves to distinguish PA and EH patients subgrouped by DM status were shown in Figure S2. The best cut-off value of ARR was 77.5 ng/dL per ng/mL/h in patients with DM, while 33.0 ng/dL per ng/mL/h in those without DM predicting PA. The ROC curves for subgroup analyses in patients subgrouped by APA and IHA were provided in the Figures S3 and S4. The results of ROC curves to confirm PA by saline infusion test (SIT) and captopril challenge test (CCT) grouped by DM status were also provided in the Figures S5–S7. 

**Figure 1:**
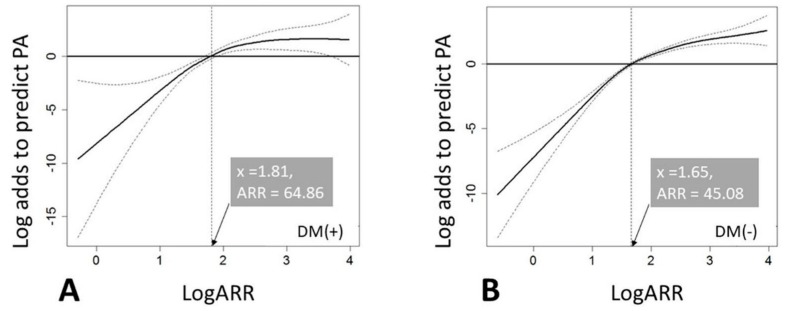
(**A**) GAM plot of screening log of the odds of the probability for PA against the log-transformed ARR in the patients with DM. The cut-off value was ARR = 64.86, (log-transformed ARR = 1.81). (**B**) GAM plot of screening log of the odds of the probability for PA against the log-transformed ARR in the patients without DM. The cut-off value was ARR = 45.08, (log-transformed ARR = 1.65). These models incorporate the subject-specific (longitudinal) random effects, expressed as the logarithm of the odd (logit). The probability of outcome events was constructed with ARR ratio and was centered to have an average of zero over the range of the data as constructed with the GAM. Abbreviations: ARR, aldosterone to renin ratio; DM, diabetes mellitus. GAM, generalised additive model; PA, primary aldosteronism.

Table 3 demonstrates the multivariable logistic regression generated for predicting the diagnosis of PA in DM patients. The cut-off value in the GAM model (Figure 1A) predicting PA among DM patients (i.e., ARR = 64.86 ng/dL per ng/mL/h; log-transformed ARR = 1.81) translated into a sensitivity of 75.0% and a specificity of 76.4%, while positive predictive value (PPV) and negative predictive value (NPV) in the study population were 82.9% and 66.7%, respectively. Likely, in patients without DM, the cut-off value in the GAM model predicting PA among non-DM patients (i.e., ARR = 45.08 ng/dL per ng/mL/h; log-transformed ARR = 1.65) (Figure 1B) translated into a sensitivity of 79.9% and a specificity of 69.6%, while PPV and NPV were 71.9% and 78.1%, respectively.

**Figure 2:**
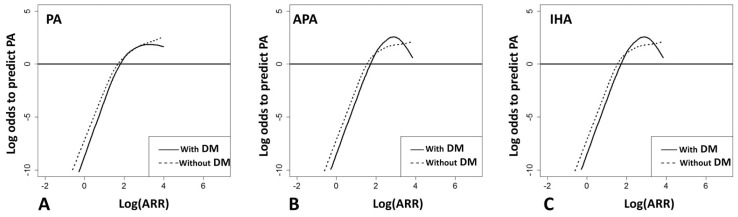
ARR to predict PA stratified by patients with or without DM. The GAM smoothing plot shows log odds to predict (**A**) PA (**B**) APA (**C**) IHA with spline Log (ARR) after multivariate adjustments. These GAM smoothing plots show a higher log-transformed ARR to predict PA in the patients with DM than those without DM in regard to log-transformed ARR < 2 (ARR < 100 ng/dL per ng/mL/h). Abbreviations: APA, aldosterone-producing adenoma; ARR, aldosterone to renin ratio; DM, diabetes mellitus; GAM, generalized additive model; IHA, idiopathic hyperaldosteronism; PA, primary aldosteronism.

**Figure 3:**
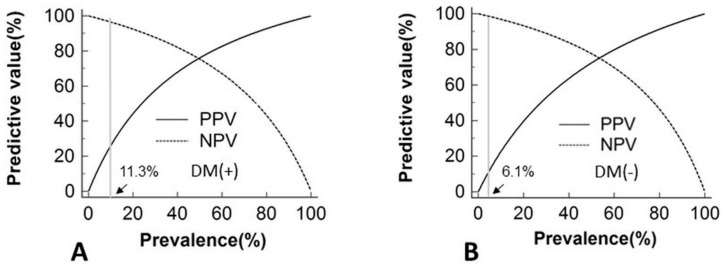
The plots demonstrate the relationship between the prevalence of PA and the diagnostic accuracy of the cut-off value as 65 ng/dL per ng/mL/h in patients with DM (**A**) and 45 ng/dL per ng/mL/h in patients without DM (**B**). The light gray line represents the reported PA prevalence of 11.3% in the hypertensive population with DM, and 6.1% in the hypertensive population without DM. Abbreviations: DM, diabetes mellitus; NPV, negative predictive value; PA, primary aldosteronism; PPV, positive predictive value.

After multivariate adjustments with age, sex, BMI, mean blood pressure, duration of hypertension, serum potassium level and eGFR, the GAM smoothing plot showed higher log-transformed ARR to predict PA in the patients with DM than those without DM, in regard to log-transformed ARR < 2 (ARR < 100 ng/dL per ng/mL/h) (Figure 2A). In line with this, we found similar results ascertained from the subgroup with APA (Figure 2B) and IHA (Figure 2C). According to the prevalence of PA, Figure 3 validated the diagnostic accuracy of the cut-off value was 65 ng/dL per ng/mL/h in DM and 45 ng/dL per ng/mL/h in non-DM patients.

## 4. Discussion

There is a higher prevalence of DM among PA patients (16%), compared with 11% among the EH patients. Thus, the comorbidity of DM will influence the timely diagnosis of aldosteronism. Patients having PA simultaneously existing with DM had higher BP and used more anti-hypertensive agents to control their BP. Our study, for the first time, illustrated that DM interferes with the screening power of ARR for diagnosing aldosteronism in diabetic and hypertensive patients. We showed that PA patients with DM are older with a longer hypertensive history than those without DM. Previous report also raised the possibility that the diagnosis of PA in hypertensive patients is more delayed in patients with DM compared to those without DM [30]. We suggest at the screening stage that DM patients have higher ARR cut-off level than patients without DM to separate PA from EH. Similar results were also validated and confirmed among APA and IHA patients. 

The RAAS, a major system that plays a pivotal role in regulating BP, electrolyte and fluid homeostasis, is easily activated in patients with DM [31,32,33]. We have found that PA patients with DM have the same serum level of PRA as PA patients without DM. Patients with DM, hypertension and nephropathy are reported to have “low renin hypertension [34]”. However, like our study, PRA in a large community sample of hypertensive patients reveals a wide distribution of activity level, especially among diabetic patients [35]. In contrast, a large study specifically addressing hypertensive subjects did not support this contention; and therefore, the anecdotal information available may have reflected highly selected diabetic patients or non-hypertensive patients with diabetes [35]. The major mechanism for this discrepancy of PRA level in our group may be volume expansion, and other mechanisms including hyalinisation of the afferent arteriole, decreased catecholamine stimulation of renin release and inadequate conversion of pro-renin to renin [34]. Furthermore, aldosterone augments fluid retention in the body by increasing salt and water retention [18]. It is also possible that our PA patients with DM may have less nephropathy; however, with hyperfiltration [16]. Similarly, age, BMI and duration of hypertension within slightly different distributions of PA patients with or without DM will determine the activity of renin. If fact, the baseline ARR ratio has been reported to be higher than 30 ng/mL/h per ng/mL/h in DM patients (even in those without hypertension) [36].

The prevalence and incidence of DM are increased in PA patients [10,11,37]. Screening for PA in type 2 DM patients with resistant hypertension is recommended for achieving BP control, and most importantly to reduce CV morbidity and mortality [10]. A meta-analysis including 4031 subjects in 16 studies reports a prevalence of 15.22% for DM in PA patients [38], very close to the 16% DM prevalence in our current PA cohort. Aldosterone can contribute to the reduction of insulin secretion indirectly via a hypokalemic effect [39]. There is a significant inverse relationship where lower potassium values are associated with higher glucose values [40]. Elevated aldosterone concentrations are associated not only with resistant hypertension but also with obesity, metabolic syndrome [41] and even with the new onset of DM [37]. Taken together, concomitant PA with DM will predispose to impaired insulin sensitivity and thus attribute to further CV events. Besides, many studies [42,43] have concluded that high levels of circulating aldosterone in heart and kidney increase local RAAS activation in brain regions that contribute to increased sympathetic tone in hypertension. In other words, the development of impaired insulin metabolic signaling and endothelial function can be promoted by excess circulating aldosterone, and sequentially contributes to hypertension and associated cardiovascular and renal structural and functional abnormalities. Therefore, it further raises the importance of diagnosing aldosteronism among patients with DM. Some investigations suggest a threshold value of PAC > 15 ng/dL and/or suppressed PRA at the screening stage to elevate the efficacy of diagnosing PA [2]. Nevertheless, the applicability varies between studies and is still controversial, even in APA or IHA. 

As for anti-diabetic drugs, since existing evidence shows very little effect on renin-angiotensin-aldosterone system (RAAS), we did not held or change any anti-diabetic medications before the ARR testing. The effect of anti-diabetic drugs on ARR, if any, remains unclear. Only pioglitazone has been reported to promote CYP11B2 expression, but nevertheless it inhibits aldosterone production in Ang II-treated HAC15 cells [44]. 15 mg pioglitazone in patients with DM complicated with coronary disease did not appear to affect the RAAS [30]. Glucagon-like peptide-1 was also reported which did not affect PRA or PAC [45].

Furthermore, as tertiary referral centers of endocrine-related hypertension, though we performed ARR testing in all diabetic patients who were referred for PA screening, we did not comprehensively screen PA in a general diabetic patient population. This is a limitation in this study. Last but not the least, screening tests are widely used in medicine to assess the likelihood that members of a defined population have a particular disease. Since the prevalence of PA in the average population is low, using ARR test for screening this diabetic population will have a high NPV but a low PPV [46]. Depending on the low prevalence rate of aldosteronism, a cut-off value of 65 ng/dL per ng/mL/h exhibits an excellent NPV in DM patients.

## 5. Conclusions

There was a considerable prevalence of PA among DM patients, which calls for prompt diagnosis of aldosteronism among DM patients. At the same time, we found no significant difference in PRA distribution between diabetic and non-diabetic PA patients. In contrast, we identified a higher cut-off value of ARR for the diagnosis of PA and suggested a better discriminative value of 65 ng/dL per ng/mL/h as a screening ARR in PA patients with DM. Before being widely applied in all PA patients with DM, prospective large-scale studies are required to confirm our results.

## Figures and Tables

**Table 1:** Clinical characteristics of 844 PA patients with DM and without DM.

General Parameters	Total, *n* = 844	DM (+), *n* = 136	DM (−), *n* = 708	*p* Value
Female (%)	455 (54)	58 (43)	395 (56)	0.010 *
BMI (kg/m^2^)	25.5 ± 4.1	26.7 ± 4.4	25.3 ± 4.0	<0.001 ^†^
Duration of HTN (years)	8.2 ± 7.8	10.9 ± 9.2	7.7 ± 7.4	<0.001 ^†^
SBP (mmHg)	149 ± 22	155 ± 22	148 ± 21	<0.001 ^†^
DBP (mmHg)	89 ± 14	90 ± 14	88 ± 14	0.196
HR (beats)	74 ± 12	74 ± 12	74 ± 12	0.673
Anti-hypertensive drugs (number)	2.3 ± 1.1	2.5 ± 1.2	2.3 ± 1.0	0.016 *
Baseline comorbidities				
CVA (%)	49 (6)	12 (9)	37 (5)	0.103
LVH (%)	128 (15)	21 (15)	107 (15)	0.917
CAD (%)	94 (11)	40 (29)	54 (8)	<0.001 ^†^
Laboratory data at screening period				
PAC (ng/dL)	55.5 ± 62.7	50.4 ± 33.3	56.5 ± 66.8	0.300
PRA (ng/mL/h)	0.53 ± 0.77	0.45 ± 0.55	0.55 ± 0.81	0.183
ARR (ng/dL per ng/mL/h)	610.9 ± 1332.7	678.9 ± 1510.0	597.8 ± 1296.7	0.516
eGFR (mL/min/1.73 m^2^)	84.5 ± 24.5	74.5 ± 26.9	86.5 ± 23.6	<0.001 ^†^
Serum potassium (mmol/L)	3.6 ± 0.7	3.6 ± 0.7	3.6 ± 0.7	0.897
24-h urinary aldosterone (μg/day)	20.2 ± 7.3	19.9 ± 8.3	20.3 ± 7.1	0.683

Data are expressed as mean ± SD or percentage. * *p* < 0.05, ^†^ *p* < 0.01. ARR, aldosterone to renin ratio; BMI, body mass index; CAD, coronary artery disease; CVA, cerebrovascular accident; DBP, diastolic blood pressure; DM, diabetes mellitus; eGFR, estimated glomerular filtration rate; HR, heart rate; HTN, hypertension; LVH, left ventricular hypertrophy; PA, primary aldosteronism; PAC, plasma aldosterone concentration; PRA, plasma renin activity; SBP, systolic blood pressure.

**Table 2:** Clinical characteristics of 225 DM patients with PA and without PA.

General Parameters	Total, *n* = 225	PA (+), *n* = 136	PA (−), *n* = 89	*p* Value
Age( years)	56.7 ± 12.3	56.1 ± 12.3	57.5 ± 12.3	0.387
Female (%)	96 (43)	58 (43)	38 (43)	0.994
BMI (kg/m^2^)	26.6 ± 4.2	26.7 ± 4.4	26.6 ± 3.9	0.884
Duration of HTN (years)	10.1 ± 8.8	10.9 ± 9.2	8.9 ± 8.2	0.034 *
SBP (mmHg)	155 ± 21	155 ± 20	154 ± 21	0.994
DBP (mmHg)	91 ± 14	92 ± 14	91 ± 15	0.528
HR (beats/min)	74 ± 11	74 ± 12	75 ± 12	0.494
Anti-hypertensive drugs (number)	2.5 ± 1.2	2.6 ± 1.2	2.5 ± 1.1	0.705
Baseline comorbidities				
CVA (%)	19 (8)	12 (9)	7 (8)	0.805
LVH (%)	28 (12)	21 (15)	7 (8)	0.100
CAD (%)	65 (29)	40 (29)	25 (28)	0.832
Laboratory data at screening				
PAC (ng/dL)	44.0 ±2 9.5	50.4 ± 33.3	34.1 ± 18.8	<0.001 ^†^
PRA (ng/mL/h)	2.0 ± 6.3	0.5 ± 0.6	4.4 ± 9.5	<0.001 ^†^
ARR (ng/dL per ng/mL/h)	474.6 ± 1296.2	678.9 ± 1510.0	162.3 ± 784.6	<0.001 ^†^
eGFR (mL/min/1.73 m^2^)	73.4 ± 27.7	74.5 ± 26.9	71.6 ± 28.9	0.453
Serum potassium (mmol/L)	3.8 ± 0.7	3.6 ± 0.7	4.2 ± 0.5	<0.001 ^†^
24-h urinary aldosterone (μg/day)	16.3 ± 7.5	19.9 ± 8.3	10.8 ± 6.2	<0.001 ^†^

Data are expressed as mean ± SD or percentage. * *p* < 0.05, ^†^ *p* < 0.01. ARR, aldosterone to renin ratio; BMI, body mass index; CAD, coronary artery disease; CVA, cardiovascular accident; DBP, diastolic blood pressure; DM, diabetes mellitus; eGFR, estimated glomerular filtration rate; HR, heart rate; HTN, hypertension; LVH, left ventricular hypertrophy; PA, primary aldosteronism; PAC, plasma aldosterone concentration; PRA, plasma renin activity; SBP, systolic blood pressure.

**Table 3:** Results of multivariable logistic regression generated for predicting the diagnosis of PA in DM patients.

General Parameters	PA (+), *n* = 136	PA (−), *n* = 89	Odds Ratio	95% CI	*p* Value
Age (years)	56.1 ± 12.3	57.5 ± 12.3			
Female (%)	58 (43)	38 (43)			
BMI (kg/m^2^)	26.7 ± 4.4	26.6 ± 3.9			
Duration of HTN (years)	10.9 ± 9.2	8.9 ± 8.2			
SBP (mmHg)	155 ± 20	154 ± 21			
DBP (mmHg)	92 ± 14	91 ± 15			
HR (beats/min)	74 ± 12	75 ± 12			
Anti-hypertensive drugs (number)	2.6 ± 1.2	2.5 ± 1.1			
Baseline comorbidities					
CVA (%)	12 (9)	7 (8)			
LVH (%)	21 (15)	7 (8)			
CAD (%)	40 (29)	25 (28)			
Preoperative laboratory data					
PAC (ng/dL)	50.4 ± 33.3	34.1 ± 18.8	1.040	1.018–1.062	<0.001 ^†^
PRA (ng/mL/h)	0.45 ± 0.55	4.4 ± 9.5	0.233	0.125–0.432	<0.001 ^†^
eGFR (mL/min/1.73 m^2^)	74.5 ± 26.9	71.6 ± 28.9			
Serum potassium (mmol/L)	3.6 ± 0.7	4.2 ± 0.5	0.196	0.098–0.394	<0.001 ^†^

Data are expressed as mean ± SD or percentage. * *p* < 0.05, ^†^ *p* < 0.01. ARR, aldosterone to renin ratio; BMI, body mass index; CAD, coronary artery disease; CVA, cerebrovascular accident; DBP, diastolic blood pressure; DM, diabetes mellitus; eGFR, estimated glomerular filtration rate; GAM, generalised additive model; HR, heart rate; HTN, hypertension; LVH, left ventricular hypertrophy; PA, primary aldosteronism; PAC, plasma aldosterone concentration; PRA, plasma renin activity; SBP, systolic blood pressure.

## Supplementary Materials

Supplementary materials can be found at http://www.mdpi.com/2077-0383/7/10/360/s1.
