# Supplementary material for: Higher Screening Aldosterone to Renin Ratio in Primary Aldosteronism Patients with Diabetes Mellitus
Source: J Clin Med. 2018 Oct 16;7(10):360. doi: 10.3390/jcm7100360 (PMC6209946; doi:10.3390/jcm7100360)
Supplement: Supplementary file 1 [file jcm-07-00360-s001.zip › Supplementary file.docx]

**Supplemental Data File**

**Higher** **Screening Aldosterone to Renin Ratio in Primary Aldosteronism Patients with Diabetes Mellitus**

**Supplemental Table 1. The comparison between PA patients and EH patients**

|  | Total, (n = 1660) | PA, (n = 844) | EH, (n = 816) | *p* value |
| --- | --- | --- | --- | --- |
| General parameters |  |  |  |  |
| Age (years) | 51.2 ± 13.8 | 52.5 ± 12.4 | 49.8 ± 14.9 | < 0.001† |
| Female (%) | 831 (50) | 455 (54) | 376 (46) | 0.001† |
| BMI (kg/m^2^) | 25.4 ± 4.0 | 25.5 ± 4.1 | 25.3 ± 4.0 | 0.372 |
| Duration of HTN (years) | 7.1 ± 7.2 | 8.2 ± 7.8 | 6.1 ± 6.4 | < 0.001† |
| SBP (mmHg) | 149 ± 21 | 149 ± 22 | 148 ± 21 | 0.211 |
| DBP (mmHg) | 89 ± 14 | 89 ± 14 | 88 ± 14 | 0.581 |
| HR (beats/min) | 74 ± 12 | 74 ± 12 | 74 ± 12 | 0.991 |
| Antihypertensive drugs (categories) | 2.1 ± 1.0 | 2.3 ± 1.1 | 2.0 ± 1.0 | < 0.001† |
| Baseline comorbidities |  |  |  |  |
| CVA (%) | 78 (5) | 49 (6) | 29 (4) | 0.031* |
| LVH (%) | 204 (12) | 128 (15) | 76 (9) | <0.001† |
| CAD (%) | 180 (11) | 94 (11) | 86 (11) | 0.696 |
| DM (%) | 225 (14) | 136 (16) | 89 (11) | 0.002† |
| Laboratory data at screening period |  |  |  |  |
| PAC (ng/dl) | 46.5 ± 48.0 | 55.5 ± 62.7 | 37.1 ± 21.5 | < 0.001† |
| PRA (ng/ml/h) | 2.5 ± 6.4 | 0.5 ± 0.8 | 4.5 ± 8.6 | < 0.001† |
| ARR (ng/dl per ng/ml/h) | 362.3 ± 1029.2 | 610.9 ± 1332.7 | 105.2 ± 434.6 | < 0.001† |
| eGFR (ml/min/1.73 m^2^) | 85.2 ± 24.4 | 84.5 ± 24.5 | 85.8 ± 24.3 | 0.280 |
| Serum potassium (mmol/L) | 3.9 ± 0.6 | 3.6 ± 0.7 | 4.2 ± 0.4 | < 0.001† |
| 24-hour urinary aldosterone (𝜇g /day) | 15.8 ± 6.0 | 20.2 ± 7.3 | 11.2 ± 4.6 | < 0.001† |

**Abbreviations:** ARR, aldosterone to renin ratio; BMI, body mass index; CAD, coronary artery disease; CVA, cerebrovascular accident; DBP, diastolic blood pressure; DM, diabetes mellitus; eGFR, estimated glomerular filtration rate; EH, essential hypertension; HR, heart rate; HTN, hypertension; LVH, left ventricular hypertrophy; PA, primary aldosteronism; PAC, plasma aldosterone concentration; PRA, plasma renin activity; SBP, systolic blood pressure. Data are expressed as mean ± SD or percentage. * p < 0.05, † p < 0.01.

**Supplemental Table 2. Clinical characteristics of 816 EH patients with DM and without DM**

|  | Total, n = 816 | DM (+), n = 89 | DM (-), n = 727 | *P* value |
| --- | --- | --- | --- | --- |
|  |  |  |  |  |
| General parameters |  |  |  |  |
| Age (years) | 49.8 ± 14.9 | 57.5 ± 12.3 | 48.9 ± 15.0 | < 0.001† |
| Female (%) | 376 (46) | 38 (43) | 338 (47) | 0.499 |
| BMI (kg/m^2^) | 25.3 ± 4.0 | 26.6 ± 3.9 | 25.2 ± 4.0 | 0.002† |
| Duration of HTN (years) | 6.1 ± 6.4 | 8.9 ± 8.2 | 5.7 ± 6.1 | < 0.001† |
| SBP (mmHg) | 148 ± 21 | 154 ± 21 | 147 ± 20 | 0.001† |
| DBP (mmHg) | 88 ± 14 | 91 ± 15 | 88 ± 13 | 0.025* |
| HR (beats/min) | 74 ± 12 | 75 ± 12 | 73 ± 12 | 0.212 |
| Antihypertensive drugs (categories) | 2.0 ± 1.0 | 2.5 ± 1.1 | 1.9 ± 0.9 | < 0.001† |
| Baseline comorbidities |  |  |  |  |
| CVA (%) | 29 (4) | 7 (8) | 22 (3) | 0.022* |
| LVH (%) | 76 (9) | 7 (8) | 69 (10) | 0.621 |
| CAD (%) | 86 (11) | 25 (28) | 61 (8) | < 0.001† |
| Laboratory data at screening period |  |  |  |  |
| PAC (ng/dl) | 37.1 ± 21.5 | 34.1 ± 18.8 | 37.4 ± 21.8 | 0.121 |
| PRA (ng/ml/h) | 4.5 ± 8.6 | 4.4 ± 9.5 | 4.5 ± 8.5 | 0.165 |
| ARR (ng/dl per ng/ml/h) | 105.2 ± 434.6 | 162.3 ± 784.6 | 98.3 ± 370.1 | 0.297 |
| eGFR (ml/min/1.73 m^2^) | 85.8 ± 24.3 | 71.6 ± 28.9 | 87.6 ± 23.1 | < 0.001† |
| Serum potassium (mmol/L) | 4.2 ± 0.4 | 4.2 ± 0.5 | 4.2 ± 0.4 | 0.824 |
| 24-hour urinary aldosterone (𝜇g /day) | 11.2 ± 4.6 | 10.8 ± 6.2 | 11.3 ± 4.4 | 0.323 |

**Abbreviations:** ARR, aldosterone to renin ratio; BMI, body mass index; CAD, coronary artery disease; CVA, cerebrovascular accident; DBP, diastolic blood pressure; DM, diabetes mellitus; eGFR, estimated glomerular filtration rate; EH, essential hypertension; HR, heart rate; HTN, hypertension; LVH, left ventricular hypertrophy; PA, primary aldosteronism; PAC, plasma aldosterone concentration; PRA, plasma renin activity; SBP, systolic blood pressure. Data are expressed as mean ± SD or percentage. * p < 0.05, † p < 0.01.

**Supplemental Figures**


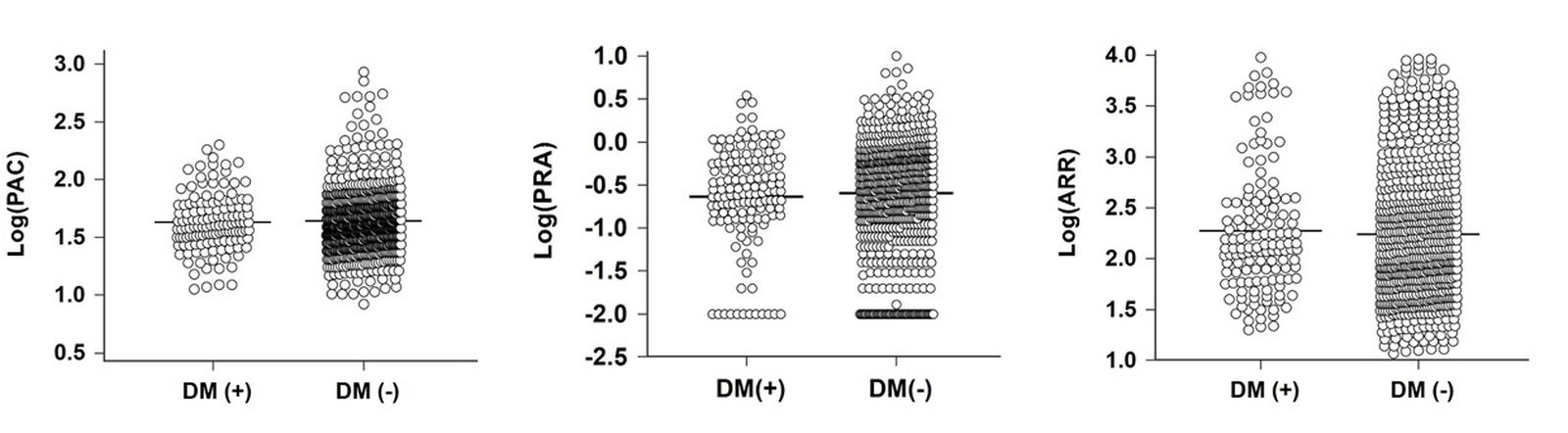


**Supplemental Fig. 1.** The dot plots show the difference of log-transformed PAC, PRA and ARR between PA patients with DM [DM (+)] and without DM [DM (-)]. The mean values of log-transformed PAC, PRA and ARR were not statistically different between DM (+) and DM (-) groups. Abbreviations: Log(PAC), log-transformed plasma aldosterone concentration; Log(PRA), log-transformed plasma renin activity; Log(ARR), log-transformed aldosterone to renin ratio; PA, primary aldosteronism; DM, diabetes mellitus.


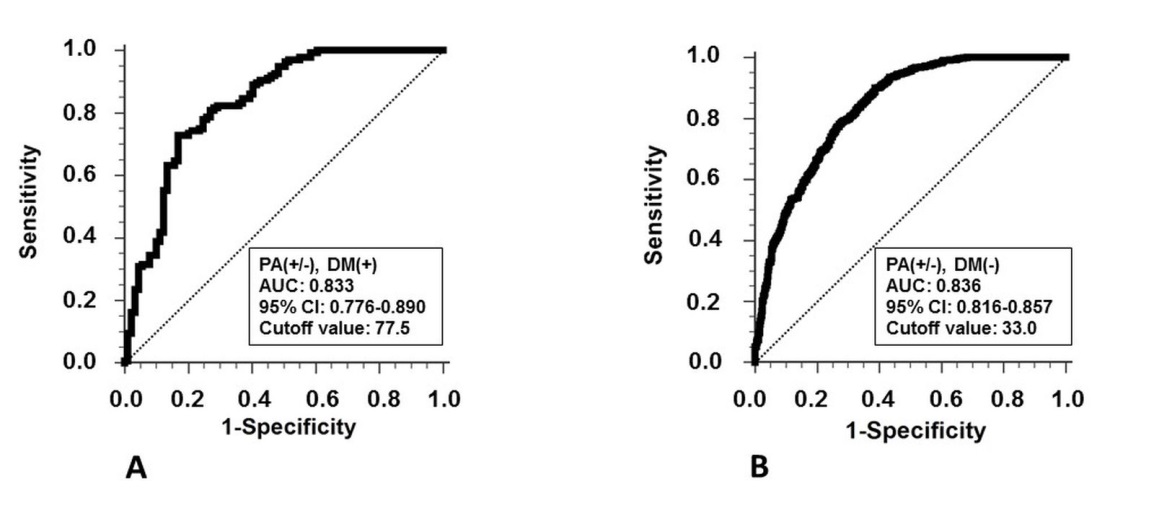


**Supplemental Fig. 2.** (A) ROC plot of screening ARR to predict PA with DM. Assuming a cut-off value of ARR ≥ 77.5 in PA patients with DM, the sensitivity is 72.8% and specificity is 83.2%. (B) ROC plot of screening ARR to predict PA without DM. The cut-off value of ARR is 33.0 in PA patients without DM, the sensitivity is 90.0% and specificity is 61.2%. Abbreviations: ROC, receiver operating characteristic; ARR, aldosterone to renin ratio; PA, primary aldosteronism; DM, diabetes mellitus; AUC, area under curve; CI, confidence interval.

**
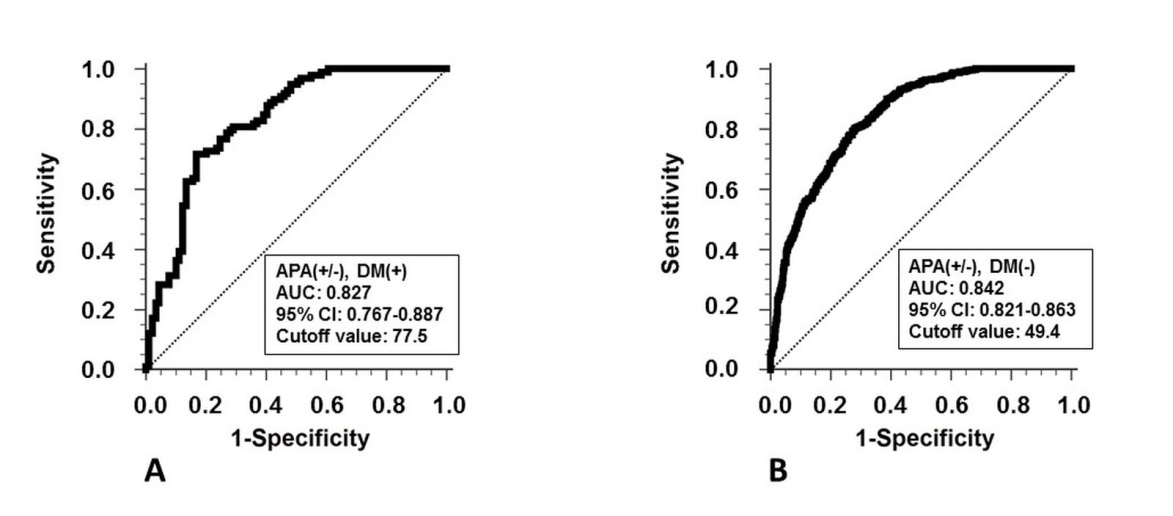
**

**Supplemental Fig. 3.** (A) ROC plot of screening ARR to predict APA with DM. Assuming a cut-off value of ARR ≥ 77.5 in APA patients with DM, the sensitivity is 71.7% and specificity is 83.2%. (B) ROC plot of screening ARR to predict APA without DM. Assuming a cut-off value of ARR ≥ 49.4 in APA patients without DM, the sensitivity is 79.3% and specificity is 72.8%. Abbreviations: ROC, receiver operating characteristic; ARR, aldosterone to renin ratio; APA, aldosterone-producing adenoma ; DM, diabetes mellitus; AUC, area under curve; CI, confidence interval.

**
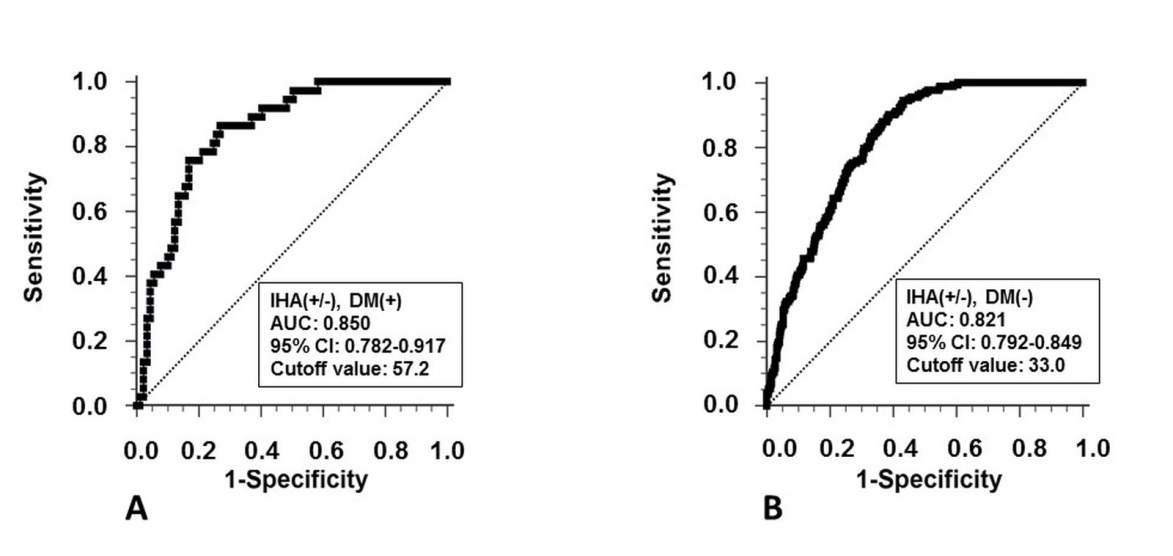
**

**Supplemental Fig. 4.** (A) ROC plot of screening ARR to predict IHA with DM. Assuming a cut-off value of ARR ≥ 57.2 in IHA patients with DM, the sensitivity is 86.5% and specificity is 73.0%. (B) ROC plot of screening ARR to predict IHA without DM. Assuming a cut-off value of ARR ≥ 33.0 in IHA patients without DM, the sensitivity is 90.1% and specificity is 61.2%. Abbreviations: ROC, receiver operating characteristic; ARR, aldosterone to renin ratio; IHA, idiopathic hyperaldosteronism; DM, diabetes mellitus; AUC, area under curve; CI, confidence interval.


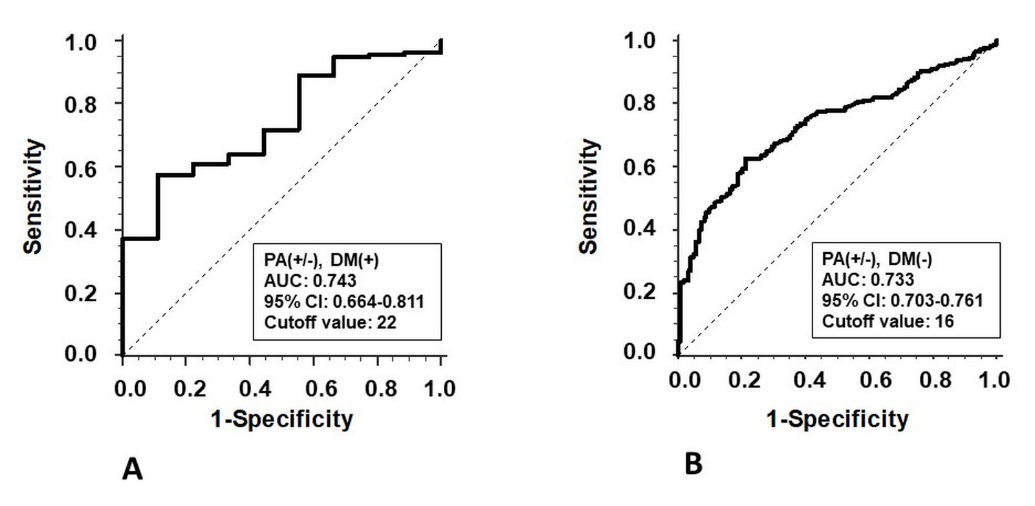


**Supplemental Fig. 5.** (A) ROC plot of aldosterone post SIT to confirm PA in DM. Assuming a cut-off value of post-SIT aldosterone ≥ 22 ng/dL in PA patients with DM, the sensitivity is 57.6% and specificity is 88.9%. (B) ROC plot of aldosterone post SIT to confirm PA in non-DM. Assuming a cut-off value of post-SIT aldosterone ≥ 16 ng/dL in PA patients without DM, the sensitivity is 62.7% and specificity is 78.7%. Abbreviations: AUC, area under curve; CI, confidence interval; DM, diabetes mellitus; PA, primary aldosteronism; ROC, receiver operating characteristic; SIT, saline infusion test.


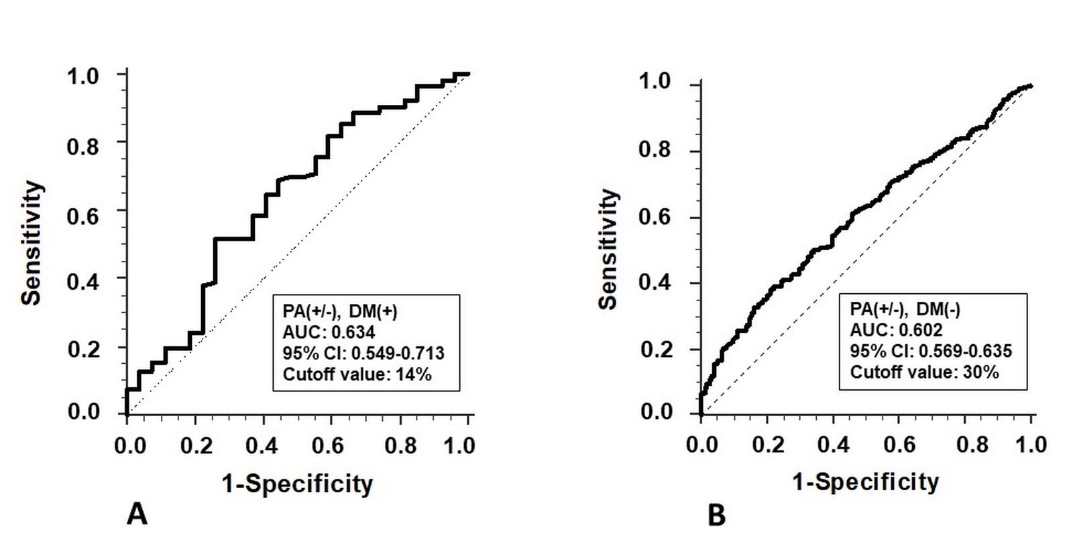


**Supplemental Fig. 6.** (A) ROC plot of the proportion of aldosterone suppression post CCT to confirm PA in DM. Assuming a cut-off value of the proportion of aldosterone suppression post CCT ≥ 14% in PA patients with DM, the sensitivity is 56.9% and specificity is 63.0%. (B) ROC plot of the proportion of aldosterone suppression post CCT to confirm PA in non-DM. Assuming a cut-off value of the proportion of aldosterone suppression post CCT ≥ 30% in PA patients without DM, the sensitivity is 44.8% and specificity is 66.2%. Abbreviations: AUC, area under curve; CI, confidence interval; DM, diabetes mellitus; PA, primary aldosteronism; ROC, receiver operating characteristic; CCT, captopril challenge test.


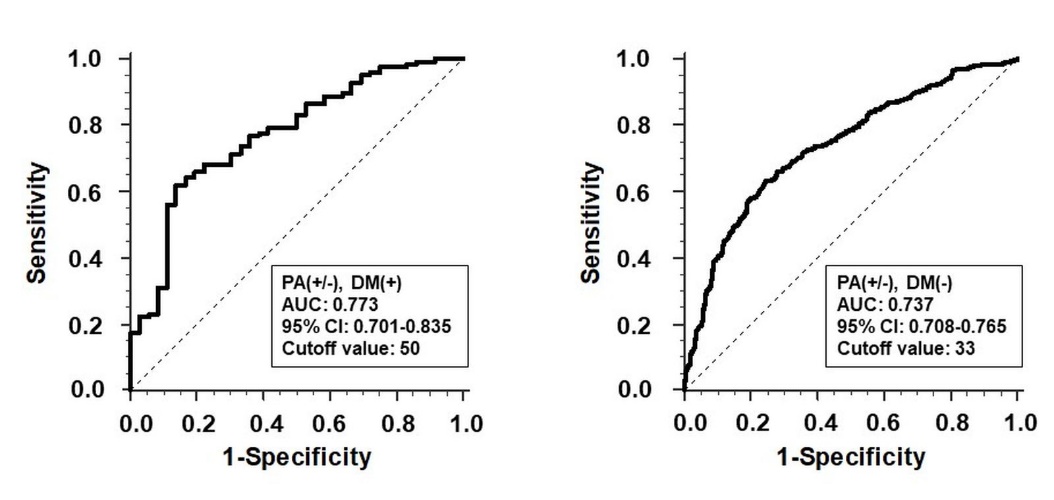


**Supplemental Fig. 7.** (A) ROC plot of the ARR post CCT to confirm PA in DM. Assuming a cut-off value of the ARR post CCT ≥ 50 ng/mL/h in PA patients with DM, the sensitivity is 61.9% and specificity is 86.1%. (B) ROC plot of the ARR post CCT to confirm PA in non-DM. Assuming a cut-off value of the proportion of aldosterone suppression post CCT ≥ 33 ng/mL/h in PA patients without DM, the sensitivity is 63.1% and specificity is 75.6%. Abbreviations: ARR, aldosterone to renin ratio; AUC, area under curve; CI, confidence interval; DM, diabetes mellitus; PA, primary aldosteronism; ROC, receiver operating characteristic; CCT, captopril challenge test.
